# C3 glomerulonephritis along with light chain proximal tubulopathy without crystal deposits in multiple myeloma: a case report

**DOI:** 10.1186/s12957-021-02135-3

**Published:** 2021-01-22

**Authors:** Junhui Xu, Xiaojuan Yu, Suxia Wang, Miao Yan, Mangju Wang, Jinping Ou, Lihong Wang, Huihui Liu, Xinan Cen

**Affiliations:** 1 Department of Hematology, Peking University First Hospital, No.8 Xi Shi Ku Street, Xi ChengDistrict, Beijing, 100034 China; 2 Department of Nephrology, Peking University First Hospital, Beijing, China; 3 Laboratory of Electron Microscopy, Pathological Centre, Peking University First Hospital, Beijing, China

**Keywords:** Multiple myeloma, C3 glomerulonephritis, Autologous stem cell transplantation, Light Chain proximal tubulopathy without crystal deposits

## Abstract

**Background:**

Multiple myeloma causes different types of renal injury. C3 glomerulonephritis (C3GN) is characterised by an abnormal deposition of complement C3 in the glomeruli due to abnormal activation of the alternative pathway of the complement system. While the association between C3GN and multiple myeloma has been well established, mild renal injury by C3GN in multiple myeloma patients with high levels of light chain has not been reported.

**Case presentation:**

A 55-year-old Chinese man presented with proteinuria. Combined with immunofixation electrophoresis, bone marrow biopsy, and renal biopsy, he was diagnosed with IgA-type multiple myeloma accompanied by C3GN and light chain proximal tubulopathy without crystal deposits. Although he had a higher level of lambda serum-free light chain, the renal injury in this patient was mild. After treatment with four courses of BD, one course of PAD, and autologous stem cell transplantation, he achieved a very good partial hematologic response with stable renal function.

**Conclusions:**

In multiple myeloma, the light chain reaches a certain level and persists, resulting in C3GN renal impairment. Early diagnosis and early intensive treatment are critical for the prognosis of such patients.

## Background

Multiple myeloma is characterised by an abnormal proliferation of bone marrow plasma cells accompanied by the excessive production of monoclonal immunoglobulin or light chain (M protein), leading to osteolytic lesions, hypercalcemia, anaemia, and kidney damage. Multiple myeloma causes different types of renal injuries [1], including cast nephropathy, amyloidosis, proliferative glomerulonephritis with monoclonal immunoglobulin deposits, immunotactoid glomerulopathy, fibrillary glomerulonephritis, and light chain proximal glomerulopathy.

C3GN is characterised by an abnormal deposition of complement C3 in the glomeruli due to abnormal activation of the alternative pathway of the complement system [2]. The most common abnormal factors that result in C3GN are autoimmune factors such as C3 nephritic factor, complement H factor antibody, properdin, and genetic variations such as mutations of complement genes coding for the components of the C3 convertase, C3, factor B, factor H, and complement factor H-related protein 5 (CFHR5), as well as copy number variations in the CFHR gene. However, C3GN has also been described in some cases of multiple myeloma [3–6]. The association between C3GN and multiple myeloma has been well established. The light chain acts as a mini-autoantibody binding within the complement regulator region of factor H, resulting in the continuous activation of the alternative pathway of the complement system and, subsequently, C3GN [7]. However, mild renal injury of C3GN in multiple myeloma patients with high levels of light chain has not been reported.

## Case presentation

A 55-year-old Chinese man presented to our centre with a 3-month history of proteinuria and bilateral eyelid oedema from 15 August 2017. This patient did not have any other significant comorbidities. His complete blood count was normal. Urinalysis showed microscopic haematuria with a red blood cell count (RBC) of 20–30/HP and proteinuria fluctuating between negative and microscale, with a 24-h urinary protein quantity (0.13 g/24 h). Biochemical tests showed normal kidney function and serum electrolyte levels, while the total protein level was decreased along with hypoalbuminemia and normal immunoglobulin level. The quantitative detection of immunoglobulin subclasses showed significant elevation of immunoglobulin A and decreased levels of immunoglobulin G and immunoglobulin M. The urine kappa light chain level was normal while that for urine lambda light chain was significantly increased (0.571 g/L, normal range < 0.005 g/L). In addition, the serum-free lambda light chain level was also greatly elevated (8.05 g/L, normal range 0.005–0.026 g/L), whereas the serum-free kappa light chain level was normal. M protein was detected by serum protein electrophoresis, while IgA-lambda-type monoclonal immunoglobulin bands were identified by immunofixation electrophoresis. Furthermore, bone marrow biopsy showed a bone marrow plasma cell (BMPC) of 42.5%. The β2-microglobulin level was slightly increased. Based on the above, we believe that this patient had IgA-lambda-type multiple myeloma (D-S: stage I group A; ISS: stage II), although bone lesions were not detected by positron emission tomography-computed tomography (PET-CT). The echocardiogram of this patient showed symmetrical thickening of the left ventricle, with thicknesses of 1.2 cm in the interventricular septum and the posterior wall of the left ventricle. Heart-enhanced MRI suggested delayed myocardial enhancement. These findings suggested cardiac amyloidosis; however, the patient refused to undergo a biopsy so the diagnosis could not be made.

**Fig. 1:**
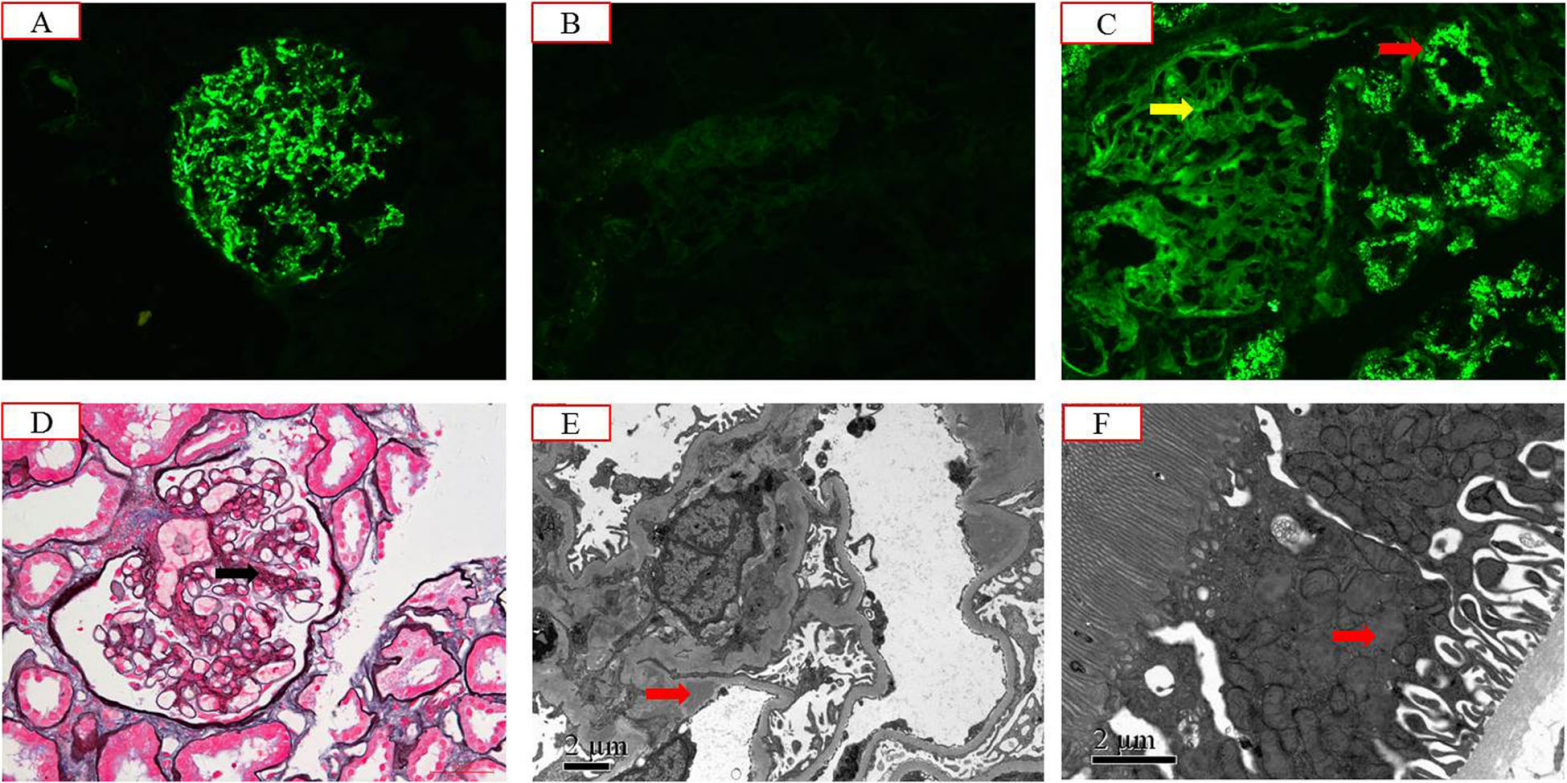
The patient’s renal pathology report showing **a** granular C3 deposition mostly in the mesangial area on the frozen section (× 200), **b** κ light chain negativity in the glomeruli and tubular epithelial cells on the frozen section (× 200), **c** stronger background staining for λ light chain than κ (yellow arrow) and massive λ reabsorption granules in the cytoplasm of tubular epithelial cells (red arrow) on the frozen section (× 200), **d** moderate mesangial proliferation (black arrow) (× 400, PASM+Masson), **e** mesangial electron-dense deposition (red arrow, × 8000), and **f** abnormal lysosomes in the cytoplasm of proximal tubular epithelial cells (red arrow, × 15,000)

To clarify the cause of renal injury in this patient, we performed a renal biopsy on 21 November 2017. Immunofluorescence microscopy showed the deposition of granular C3 in the mesangial area (Fig. 1a). Kappa light chain staining was negative (Fig. 1b). Granular deposition of the lambda light chain was observed in the renal tubular epithelium (Fig. 1c). Under light microscopy, glomerular mesangial cell hyperplasia was noted (Fig. 1d). Mild segmental hyperplasia of the stroma with a small amount of eosinophil deposition, granular degeneration of renal tubular epithelium, and renal interstitium with small focal lymphocytes and infiltrating monocytes were also detected. Additionally, electron microscopy showed dense deposits in the mesangial areas (Fig. 1e). Abnormal lysosomes in the cytoplasm of proximal tubular epithelial cells were also noted (Fig. 1f). The patient had marked hypocomplementemia with reduced complement C3 level, but normal complement C4 level. In addition, his levels of C3 nephritic factor, complement factor H antibody, complement factor H concentration, and vWF-cleaving protease (ADMAMTS13) activity were also normal.

**Fig. 2:**
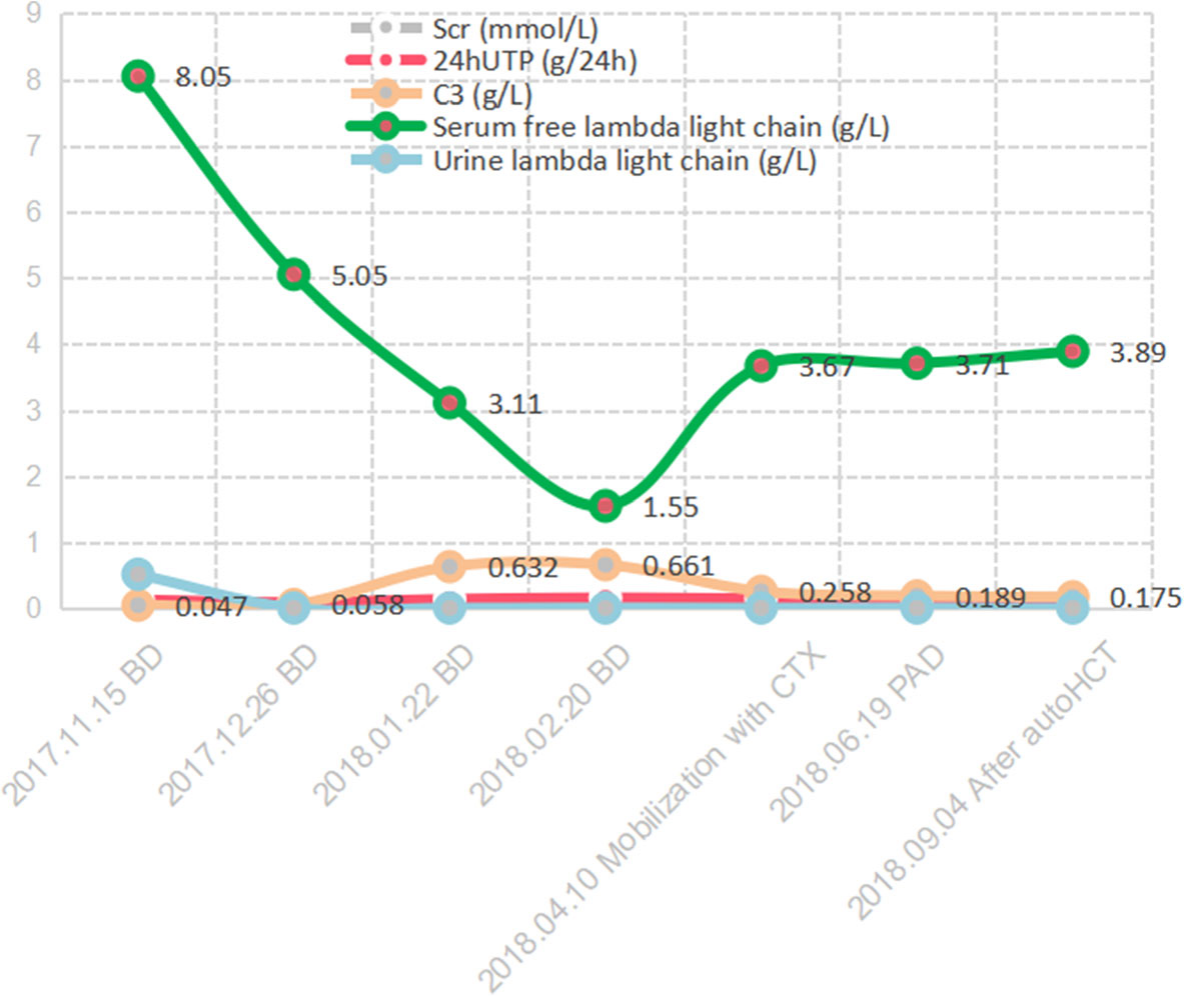
The timeline of kidney function, 24 h UTP, C3, serum-free lambda light chain, and urine lambda light chain levels relative to treatment. BD, bortezomib plus dexamethasone; CTX, cyclophosphamide; PAD, bortezomib plus doxorubicin and dexamethasone; autoHCT, autologous stem cell transplantation

The patient was diagnosed with C3GN along with light chain proximal tubulopathy without crystal deposits resulting from multiple myeloma. The patient showed a very good response to four courses of bortezomib plus dexamethasone (BD) treatment. Chemotherapy was suspended due to the need to mobilise and collect autologous stem cells; however, the efficiency of mobilisation and collection was poor and required significant time. On 19 April 2018, the patient showed a disease relapse. He was then treated with one course of bortezomib plus doxorubicin and dexamethasone (PAD). To further consolidate the therapeutic effect, a BEAM (bis-chloroethylnitrosourea [BCNU], etoposide, cytarabine, and melphalan) pretreatment protocol was administered for 6 days, followed by autologous stem cell transplantation on 11 July 2019. The patient again achieved a very good partial response with stable renal function. The timeline of kidney function, proteinuria, C3, serum-free lambda light chain, and urine lambda light chain levels relative to treatment are shown in Fig. 2 and Table 1. The patient then went to another hospital for treatment and died of cardiac arrest on 13 May 2020.

**Table 1:** The timeline of clinical indicators levels relative to treatment

Time	Scr (mmol/L)	24 h UTP (g/24 h)	C3 (g/L)	Serum-free lambda light chain (g/L)	Urine lambda light chain (g/L)
2017.11.15 BD	0.0606	0.13	0.047	8.05	0.517
2017.12.26 BD	0.0541	0.09	0.058	5.05	0.005
2018.01.22 BD	0.0563	0.14	0.632	3.11	0.005
2018.02.20 BD	0.0581	0.16	0.661	1.55	0.005
2018.04.10 CTX^*^	0.0605	0.15	0.258	3.67	0.005
2018.06.19 PAD	0.0668	0.16	0.189	3.71	0.005
2018.09.04 after autoHCT	0.0572	0.14	0.175	3.89	0.005

*Abbreviations*: *Scr* serum creatinine, *24 h UTP* 24-h urinary protein quantity, *BD* bortezomib plus dexamethasone, CTX^*^ mobilisation with cyclophosphamide, *PAD* bortezomib plus doxorubicin and dexamethasone, *autoHCT* autologous stem cell transplantation

## Discussion

C3GN is mainly caused by abnormal factors involved in the alternative pathway of the complement system. Previous studies found that the light chain acted as a mini-autoantibody binding within the complement regulator region of factor H, resulting in the continuous activation of the alternative pathway of the complement system and, ultimately, C3GN [7]. In our case, the levels of C3 nephritic factor, complement factor H antibody, and complement factor H concentration were normal. During treatment, there was a negative correlation between serum-free lambda light chain and complement C3 levels (Fig. 2), indicating that the light chain was a key factor in complement system activation.

Drayson and Karatoy Erdem reported a strong correlation between higher levels of serum-free light chains (> 800 mg/dL) and the severity of multiple myeloma renal impairment [8, 9]. While varying degrees of renal injury was observed in six patients previously reported, our patient, who had a higher lambda serum-free light chain level, did not show a significant increase in serum creatinine and 24hUTP levels. There are two explanations for this situation. First, although our patient had a higher serum-free light chain level, the time from onset to diagnosis was too short to result in renal dysfunction. Second, after our patient received treatment, the serum-free light chain level rapidly fell below the threshold, resulting in renal dysfunction. Therefore, the light chain reaches a certain level and persists, leading to renal impairment of C3GN in multiple myeloma. The threshold light chain level for renal impairment of C3GN requires further investigation.

An early diagnosis of C3GN by renal biopsy can shorten the duration of high-level light chains before diagnosis. In addition, early diagnosis of C3GN by renal biopsy can allow early treatment to further shorten this duration. C3GN may affect the survival time of patients with multiple myeloma; therefore, early diagnosis of C3GN by renal biopsy in multiple myeloma is particularly important. Thus, patients with renal injury in multiple myeloma with low complement C3 levels should undergo renal biopsy to determine the type of renal pathology. As the light chain level is related to the disease severity, we also recommend more aggressive treatment plans in the early stage to reduce light chain levels.

## Conclusions

In summary, the light chain reaches a certain level and persists, causing renal impairment of C3GN in multiple myeloma. Early diagnosis and intensive treatment are critical for the prognosis of such patients.

## Data Availability

All data generated or analysed during this study are included in this published article.
